# IL-10/STAT3/SOCS3 Axis Is Involved in the Anti-inflammatory Effect of Benznidazole

**DOI:** 10.3389/fimmu.2019.01267

**Published:** 2019-06-04

**Authors:** Ágata C. Cevey, Federico N. Penas, Catalina D. Alba Soto, Gerardo A. Mirkin, Nora B. Goren

**Affiliations:** ^1^Facultad de Medicina, Instituto de Investigaciones Biomédicas en Retrovirus y SIDA (INBIRS), CONICET—Universidad de Buenos Aires, Buenos Aires, Argentina; ^2^Facultad de Medicina, Instituto de Investigaciones en Microbiología y Parasitología Médica (IMPaM), CONICET—Universidad de Buenos Aires, Buenos Aires, Argentina

**Keywords:** benznidazole, anti-inflammatory effects, cardiomyocytes, mechanism of action, inflammatory mediators

## Abstract

Anti-parasitic treatment for Chagas disease mainly relies on benznidazole, which is virtually the only drug available in the market. Besides its anti-parasitic effects, benznidazole has anti-inflammatory properties. In this work we studied the mechanisms involved in the latter, demonstrating the participation of the IL-10/STAT3/SOCS3 pathway. To achieve this goal, the anti-inflammatory properties of benznidazole were studied using an *in vitro* model of cardiomyocyte primary culture stimulated with LPS. LPS increased both SOCS3 expression and STAT3 phosphorylation. The addition of benznidazole increased their expression even further. Specific inhibition of STAT3 precluded this effect, suggesting a role for STAT3 in the increase of SOCS3 expression induced by benznidazole. To assess the participation of SOCS3 in the anti-inflammatory effect of benznidazole, we accomplished specific knockdown of SOCS3 with siRNA. Silencing of SOCS3 in cardiomyocytes precluded the inhibitory effects of benznidazole on TNF-α, IL-6, iNOS expression and NO release. Moreover, in the absence of SOCS3, benznidazole could neither prevent IKK phosphorylation nor IκBα degradation, supporting the notion that SOCS3 is required for the benznidazole-mediated inhibition of the NF-κB pathway. Previously, we demonstrated that IL-10 increases the expression of SOCS3 in cultured cardiomyocytes. Here, we found that benznidazole shows a trend to increased IL-10 expression. To evaluate whether benznidazole increased SOCS3 in an IL-10-dependent manner, cardiomyocytes from IL-10 knockout mice were pre-treated with benznidazole and stimulated with LPS. Benznidazole neither inhibited NO release nor avoid IKK phosphorylation or IκBα degradation, showing that IL-10 is required for benznidazole-mediated inhibition of NF-κB. Moreover, exogenous addition of IL-10 to IL-10 knockout cardiomyocytes restored the inhibitory effect of benznidazole on NO release. The results reported herein show, for the first time, that the IL-10/STAT3/SOCS3 axis is involved in the anti-inflammatory effects of benznidazole. These findings may add up to new therapeutic strategies for chronic Chagas disease given its inflammatory nature.

## Introduction

American trypanosomiasis is an endemic parasitic disease caused by infection with the obligate intracellular protozoan parasite *Trypanosoma cruzi* (*T. cruzi*). It spans throughout Central and South America and represents a major public health concern due to its high morbidity and mortality rates (1). Benznidazole (*N*-benzyl-2-(2-nitroimidazol-1-yl)acetamide) is one of the drugs of choice for its treatment (2) despite its toxicity and limited efficacy, particularly during the chronic phase of the disease. Although benznidazole has long been used in clinical settings, its mechanisms of action have not been fully elucidated yet. Indeed, there is a general premise that the etiological treatment contributes to a reduction of the parasite load and to fit the host immune response, leading to a balanced inflammatory response which is crucial to control Chagas disease morbidity (3, 4). In a previous work, Campi-Azevedo et al. highlighted that treatment with benznidazole in the indeterminate phase of the disease not only leads to parasite clearance but also would contribute to a general immunomodulation. Induction of a broad change in the immune response was observed in patients in the cardiac phase, shaping an intricate phenotypic/functional network compatible with beneficial and protective immunological events (5). In this regard, we have recently shown in an experimental murine model of acute Chagas disease, using a highly virulent benznidazole-susceptible *T. cruzi* strain, that the optimal effects of benznidazole, in terms of parasite clearance from blood and heart tissue, as well as the reduction of inflammatory reaction, can be achieved at doses significantly lower than those usually used for the treatment (6).

In addition to its antiparasitic activity, benznidazole exerts immunomodulatory effects on macrophages stimulated with lipopolysaccharide (LPS) and treated with a high concentration of benznidazole (1 mM), by inhibiting the NF-κB pathway (7). These *in vitro* effects have also been described in LPS-challenged mice pre-treated with high doses of benznidazole (200 mg/Kg/day) (8). Like LPS, certain *T. cruzi* components, such as glycoinositolphospholipids, are recognized by TLR4 and induce pro-inflammatory cytokines. Thus, in order to distinguish the anti-inflammatory effects from the anti-parasitic effects of benznidazole, we used LPS in experimental settings aimed at exploring its anti-inflammatory mechanism of action (9, 10). Interestingly, anti-inflammatory as well as anti-parasitic effects were achieved using 15 μM of benznidazole, a concentration rendering parasite DNA almost undetectable by qPCR (6).

Interleukin-10 (IL-10), a well-known anti-inflammatory cytokine, is produced by a range of cells such as T cells, B cells, macrophages, and dendritic cells. Its expression is regulated by multiple signaling molecules, including p38 MAPK and ERK1/2 (11). One of the main biological function of IL-10 is to counter the production of inflammatory mediators, especially in response to TLR signaling (12–16). The binding of IL-10 to the IL-10R results in the activation of JAK1 which induces STAT3 phosphorylation. It has been demonstrated that STAT3 is a key effector molecule of IL-10 action. Its activation is necessary for the IL-10-regulated anti-inflammatory effects (17–20). Although better studied in macrophages (17, 21–26), the IL-10/STAT3 anti-inflammatory pathway has long been known to extend to other cells of the immune system (19, 27–30), and non-immune cells (31–35).

Both IL-10 and Interleukin-6 (IL-6) induce the activation of STAT3, yet generate different cellular responses. While IL-6 stimulation promotes a pro-inflammatory response, IL-10 signaling induces a strong anti-inflammatory one (36). Activated STATs not only drive transcription of many genes related to cell proliferation, function, and survival, but also induce the transcription of SOCS genes (37). SOCS3, one of the better studied members of the SOCS family, controls critical cellular processes such as cell growth, apoptosis, and transcription of inflammatory genes (38). It also regulates the kinetics of STAT3 activation, determining the pattern of responsive genes in the case of IL-6 and IL-10 (36).

Induction of SOCS3 by STAT3 results in a negative feedback loop, *via* binding to the gp130 subunit of the IL-6 receptor, which results in transient STAT3 activation with a rapid decline in phosphorylation and nuclear localization. In contrast, SOCS3 does not block IL-10 activation of STAT3, thereby inducing sustained STAT3 activation (39, 40). This confers a particular timing to the initiation and perpetuation of the inflammatory response.

We have described that 15 μM of benznidazole has the ability to inhibit the NF-κB pathway, demonstrating that the anti-inflammatory effects of this drug may be attained at a concentration lower than that reported elsewhere (6).

The aim of this work is to deepen into the mechanisms involved in the anti-inflammatory effects of benznidazole, as they have not yet been fully elucidated. Our results show that benznidazole leads to STAT3 activation and up-regulation of SOCS3. Moreover, we demonstrate for the first time that IL-10 is required for the benznidazole-mediated inhibition of NF-κB, through IL-10/STAT3/SOCS3 axis.

## Materials and Methods

### Ethics Statement

To carry out this work, CF1, BALB/c, and BALB/c-background IL-10 knockout mice, homozygous for the targeted mutation Il10tm1Cgn (Stock Number 004333; The Jackson Laboratory, USA) (41) were used. All the animals were bred and maintained in the animal facility at the Instituto de Investigaciones en Microbiología y Parasitología Médica, Universidad de Buenos Aires—CONICET. All the procedures were approved by the Institutional Committee for the Care and Use of Laboratory Animals (CICUAL, Facultad de Medicina de la Universidad de Buenos Aires, CD N° 2271/2014), in line with guidelines of the Argentinean National Administration of Medicines, Food, and Medical Devices (ANMAT), Argentinean National Service of Agri-Food Health and Quality (SENASA) and with the Guide for the Care and Use of Laboratory Animals (NIH, USA).

### *In vitro* Model: Neonatal Mouse Primary Cardiomyocytes Culture and Stimulation

For each experiment, 20–30 neonatal mice (One- to three-day old, male, and female, 2–3 g weight) were euthanized by decapitation after CO_2_ exposure, and cardiomyocytes were obtained as described previously (6). Briefly, the hearts were removed aseptically, pooled and maintained in Hank's Buffer Saline Solution (HBSS). The hearts were mechanically disaggregated, and the cells were isolated after several digestions with porcine pancreas trypsin (0.25% w/v in PBS). The cells were plated in culture plates in order to generate five replicates *per* experimental group, with complete medium (10% FBS-DMEM-M199-PenStrep®) at 37°C in a 5% CO_2_ atmosphere, up to 80% confluence. Then, the cells were maintained with 1% FBS-DMEM-M199 -PenStrep®, at least for a day before the experiments were performed. For SOCS3 silencing experiments using SOCS3 siRNA and oligofectamine®, cells were cultured up to 30% confluence with complete medium, and maintained with DMEM-M199 medium (without FBS and PenStrep®), at least for a day before stimulation.

#### *In vitro* Treatments

**Figure 1:**
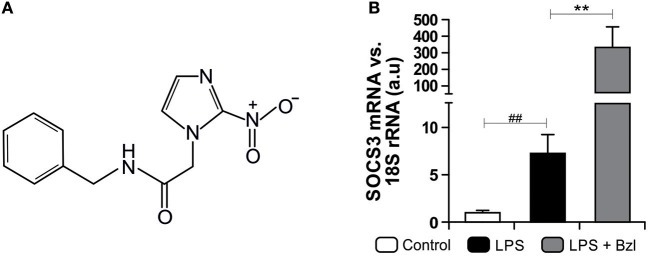
The chemical structure of benznidazole (Bzl) used in this study is shown **(A)**. Cardiomyocytes were treated with 15 μM of Bzl for 30 min and then with 10 mg/L LPS for 24 h. SOCS3 mRNA levels were analyzed by RT-qPCR and normalized against 18S rRNA **(B)**. Results are expressed as the mean of three independent experiments (*n* = 3, 5 replicates/treatment) ± SEM. White bar: Unstimulated control cells. Black bar: LPS stimulated cells. Gray bar: LPS stimulated and Bzl treated cells **P < 0.001, vs. LPS-stimulated cells; ^##^*P* < 0.001 vs. control cells.

Benznidazole (Abarax®, ELEA, Argentina. PubChem Compound Database CID = 31,593, Figure 1A) was suspended in PBS. According to the experiment, stimulated cells were pre-treated with benznidazole 15 μM for 30 min before stimulation with LPS in DMEM.

Recombinant Mouse Interleukin-10 (rm IL-10, Immunotools Cat. #12340102) was suspended in PBS. Cardiomyocytes were treated with 50 ng/mL of rmIL-10 for 30 min before benznidazole treatment and LPS stimulation.

#### *In vitro* Stimulation

The cardiomyocytes were stimulated with LPS (10 μg/ml) from *Escherichia coli* O26:B6 (Sigma-Aldrich Co, USA), for the indicated period of time, in polystyrene culture plates. Each independent experiment was carried out three times, with five replicates *per* experimental group each one.

#### STAT3 Inhibition

Cardiomyocytes were treated with 10 μM of the specific STAT3 Inhibitor V, Stattic (Santa Cruz Biotechnology, USA), 30 min before benznidazole treatment and LPS stimulation. Stattic inhibits the activation of the STAT3 transcription factor by blocking phosphorylation and dimerization events (31, 42). Briefly, Static was re-suspended in DMSO to generate a 50 mM stock solution. Working solution (500 μM) was generated diluting the stock solution in PBS (Final concentration of DMSO: 1%). PBS/DMSO was added to each untreated well in order to perform vehicle controls (Final concentration of DMSO, 1% in PBS).

#### SOCS3 Knockdown With Small Interfering RNA (siRNA)

Cardiomyocytes were cultured up to 30–50% of confluence in DMEM: M-199 medium containing 5% FCS without antibiotics for 24 h. Thereafter, cells were transfected with 20 μM of SOCS3-BLOCKiT™siRNA that targets SOCS3 mRNA (siRNA duplex: 5′-GUAUGAUGCUCCACUUUAATT-3′, 5′-UUAAAGUGGAGCAUCAUACTT-3′), following the manufacturer's instructions (Life Technologies Inc., USA). Transfections were performed with Oligofectamine (Life Technologies, Inc., USA) as specified by the manufacturer. Assays for gene activity were performed at 72 h post-transfection. The impact of SOCS3-siRNA interference on SOCS3 mRNA was evaluated by RT-qPCR.

### RNA Purification

Total RNA was obtained from cardiomyocytes using Quickzol reagent (Kalium Technologies, Argentina), treated with RQ1 RNase-Free DNase (Promega Co., USA). Total RNA was reverse-transcribed using M-MLV Reverse Transcriptase (Promega Co., USA), according to manufacturer's instructions.

### Quantitative Reverse Transcription Polymerase Chain Reaction (RT-qPCR)

mRNA expression was determined using 5X HOT FIREPOL EVAGREEN qPCR (Solis BioDyne, Estonia) in a StepOnePlus Real-Time PCR System. Parameters were: 52°C for 2 min, 95°C for 15 min, and 40 cycles at 95°C for 15 s, specific Tm °C for 30 s and 72°C for 1 min. Normalization was carried out using 18S rRNA. Quantification was performed using the comparative threshold cycle (Ct) method, as all the primer pairs (target gene/reference gene) were amplified using comparable efficiencies (relative quantity, 2^−ΔΔCt^) (43, 44).

#### Primer Sequences

**Table:** 

18S:	Fw: 5′AACACGGGAAACCTCACCC3′;	
	Rv: 5′CCACCAACTAAGAACGGCCA3′;	Tm: 60°C
IL-6:	Fw: 5′TGATGCACTTGCAGAAAACAA3′;	
	Rv: 5′GGTCTTGGTCCTTAGCCACTC3′;	Tm: 60°C
TNF-α:	Fw: 5′CGGGCAGGTCTACTTTGGAG3′;	
	Rv: 5′ACCCTGAGCCATAATCCCCT3′;	Tm: 62°C
iNOS:	Fw: 5′CACAGCAATATAGGCTCATCCA3′;	
	Rv: 5′GGATTTCAGCCTCATGGTAAAC3′;	Tm: 60°C
SOCS3:	Fw: 5′CCTTTGACAAGCGGACTCTC3′;	
	Rv: 5′GCCAGCATAAAAACCCTTCA3′;	Tm: 60°C
IL-10:	Fw: 5′CTCCCCTGTGAAAATAAGAGCA3′;	
	Rv: 5′TCCAGCAGACTCAATACACACT3′;	Tm: 60°C
IL-10R:	Fw: 5′-TGTTTACTTATCACGACGGA3′;	
	Rv: 5′GACCAGGACTGTAGGCAACTT3′;	Tm: 56°C

### Cytokine ELISA

IL-6, TNF-α, and IL-10 concentrations were measured in culture supernatants using cytokine-specific OptEIA™ ELISA kits according to the manufacturer's instructions (BD Biosciences, USA). The reaction was detected using peroxidase-conjugated Streptavidin, followed by incubation with hydrogen peroxide as a substrate, and with ABTS (Sigma Aldrich Co., USA) as a chromogen. Cytokine concentrations in samples were interpolated from recombinant IL-6, TNF-α, and IL-10 standard curves. Absorbance readings were made at 405 nm.

### Protein Extraction and Western Blot Analysis

Total protein extracts were prepared as described previously by our group (6). Protein concentration was determined by the Bradford method using a commercial protein assay (Bio-Rad, USA) and bovine serum albumin (BSA, Sigma-Aldrich Co, USA.) as a standard (45).

Fifty microgram of protein extracts separated in 8–15% SDS-PAGE gels were blotted onto a Hybond-P membrane (GE Health-care, Spain) to detect SOCS3 (Elabscience®, Cat# E-AB-10603, USA), p-STAT3 (Tyr705) (BioLegend Antibody Cat# 690402, RRID:AB_2629819, USA), and α-actin (Santa Cruz Biotechnology Cat# sc-1615, RRID:AB_630835, USA), using specific antibodies, in a 1:500 dilution in PBS. Blots were revealed by enhanced chemiluminescence in a BioSpectrum® Imaging Systems (UVP, Analytik Jena Company, USA). Band intensity was analyzed using the NIH Image J software (ImageJ, RRID:SCR_003070).

### NO Measurement

To determine the amount of NO released into the culture medium, nitrate was reduced to nitrite and measured spectrophotometrically by the Griess reaction (46, 47). The amount of NO in the culture supernatants was calculated by interpolation of the samples absorbances at 540 nm using a standard curve of NaNO_2_.

### Immunofluorescence

Myocardial cells grown on round glass coverslips were fixed with methanol and blocked with 3% BSA in PBS. The expression of p-IKK and IκBα was determined by immunofluorescence, as described previously (6). For this purpose, rabbit polyclonal IgG anti- IκBα (Santa Cruz Biotechnology Cat# sc-1643, RRID:AB_627772) or rabbit polyclonal IgG anti- p-IKK (Ser176) (Santa Cruz Biotechnology Cat# sc-21661, RRID:AB_669364) were used as primary antibodies at a 1:50 dilution, and Goat anti-rabbit IgG Alexa Fluor 488 nm (Jackson ImmunoResearch Labs Cat# 111-545-003, RRID:AB_2338046), or Goat anti-Rabbit IgG Alexa Fluor 594 nm (Jackson ImmunoResearch Labs Cat# 111-585-003, RRID:AB_2338059) were used at a 1:500 dilution as secondary antibodies. Cells nuclei were stained with DAPI (300 nM). At least 30 random microscopic fields (400X) and 1,000 cells *per* culture were acquired using a Spot RT digital camera attached to an Eclipse 600 fluorescence microscope (Nikon Inc., USA). Mean fluorescence intensity (MFI) were quantified using Fiji Image J software (Fiji, RRID:SCR_002285) (48).

### Statistical Analysis

Data are expressed as the mean of three independent experiments ± SEM (*n* = 3) for each experimental group (Five culture replicates/group). One-way ANOVA was used to analyze the statistical significance of the differences observed between infected, treated or untreated groups. The Tukey *post-hoc* test was performed to compare every mean with every other mean. Differences were considered statistically significant when *P* < 0.05. All analyses were performed using the Prism 7.0 Software (GraphPad Prism, RRID:SCR_002798).

## Results

### LPS and Benznidazole Promote STAT3 Activation and SOCS3 Expression

In a previous work we found that *T. cruzi* and IL-10 promote STAT3 phosphorylation and up-regulate the expression of SOCS3 thereby preventing NF-κB nuclear translocation and ERK1/2 phosphorylation (31). Besides, we determined that the treatment with benznidazole 15 μM was the optimal concentration to clear parasite load in *T. cruzi*-infected primary cultured cardiomyocytes, as measured by qPCR (6). These findings led us to study its anti-inflammatory properties given the optimal parasiticidal concentration found. We determined that benznidazole 15 μM reduced inflammatory mediators via inhibition of the NF-κB pathway (6). In this work, we sought to determine whether SOCS3 was involved in the anti-inflammatory effects of benznidazole. To this aim, we set up a model to dissociate the anti-parasitic effect of benznidazole from its anti-inflammatory properties using LPS instead of live parasites. Cultured cardiomyocytes were pre-treated with 15 μM benznidazole and stimulated with 10 mg/L of LPS. Interestingly, an increase in SOCS3 mRNA level was observed 24 h after LPS stimulation. Moreover, pre-treatment with benznidazole further increased SOCS3 expression significantly, as shown in Figure 1B.

**Figure 2:**
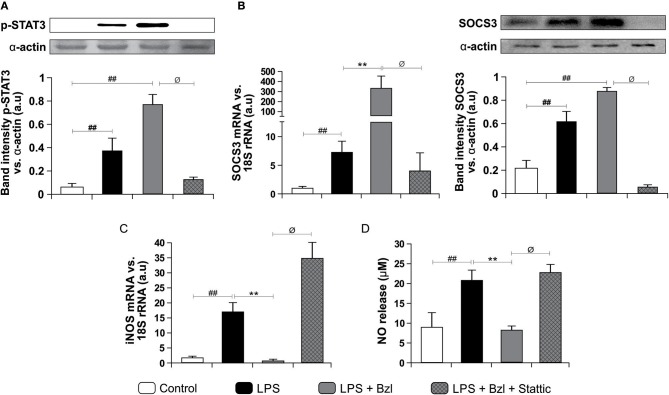
Cardiomyocytes were treated with 10 μM Stattic, a specific inhibitor of STAT3, 30 min before the addition of 15 μM benznidazole (Bzl). Cells were stimulated with 10 mg/L LPS 30 min after Bzl treatment. After 15 min, p-STAT expression was determined by Western blot with a specific antibody and normalized against α-actin **(A)**. After 24 h, SOCS3 mRNA levels were analyzed by RT-qPCR and normalized against 18S rRNA. After 48 h SOCS3 protein expression was determined by Western blot with a specific antibody and normalized against α-actin **(B)**. After 48 h, iNOS mRNA levels were analyzed by RT-qPCR and normalized against 18S rRNA **(C)**, and NO levels were quantified by the Griess reaction in culture supernatants **(D)**. Results are expressed as the mean of three independent experiments (*n* = 3, 5 replicates/treatment) ± SEM. White bar: Unstimulated control cells. Black bar: LPS stimulated cells. Gray bar: LPS stimulated and Bzl treated cells. Hatched gray bar: Stattic pre-treated, LPS stimulated and Bzl treated cells **P < 0.001, vs. LPS-stimulated cells; ^##^*P* < 0.001 vs. control cells.^ø^*P* < 0.05 vs. Bzl-treated and LPS-stimulated cells.

Moreover, both LPS and benznidazole are able to induce phosphorylation of STAT3. As expected, this effect was inhibited by 10 μM of Stattic, a STAT3 inhibitor (Figure 2A). Interestingly, SOCS3 increase was precluded by Stattic, suggesting that benznidazole increased SOCS3 mRNA expression in a STAT3-dependent manner (Figure 2B). As expected, Western blot showed an increase in SOCS3 protein expression after LPS stimulation and even more in LPS-stimulated and benznidazole-treated cardiomyocytes. Furthermore, lower SOCS3 expression upon treatment with Stattic of LPS-stimulated and benznidazole-treated cardiomyocytes was observed (Figure 2B).

Earlier, we reported that while LPS increases the expression of iNOS and the release of NO by cardiomyocytes, benznidazole inhibits these responses (6). In this work we assessed the participation of STAT3 in the inhibitory effect of benznidazole on these inflammatory mediators. When STAT3 is inhibited, benznidazole neither prevents iNOS expression (Figure 2C) nor NO release (Figure 2D), pointing out that STAT3 is involved in the anti-inflammatory effect of benznidazole.

### SOCS3 Is Involved in the Anti-inflammatory Effects Exerted by Benznidazole

**Figure 3:**
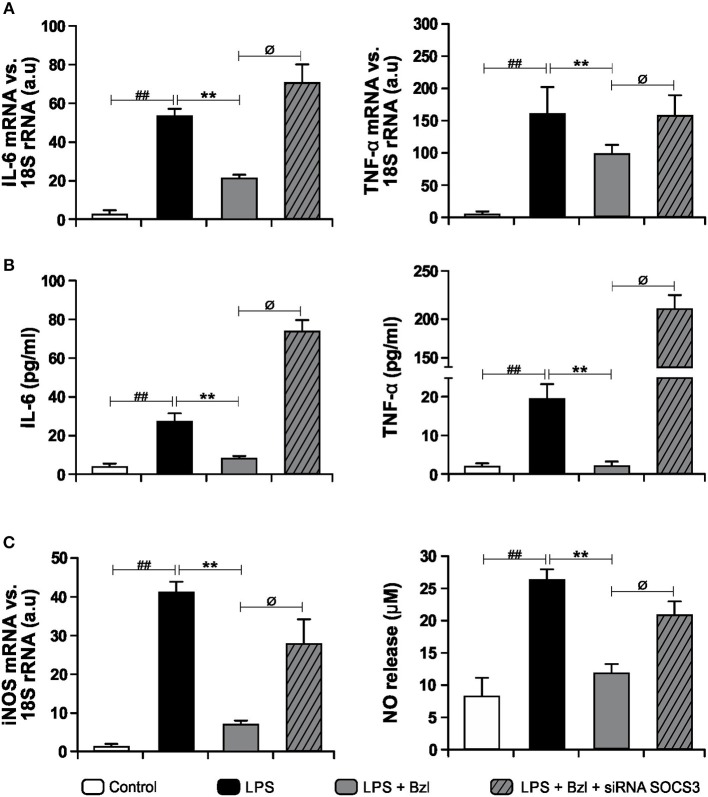
Cardiomyocytes were transfected with SOCS3 siRNA. After 72 h, cells were treated with 15 μM benznidazole (Bzl) for 30 min and then with 10 mg/L LPS. After 4 h, IL-6 and TNF-α mRNA levels were analyzed by RT-qPCR and normalized against 18S rRNA **(A)**. Concentration of cytokines in culture supernatants was measured by ELISA **(B)**. After 48 h, iNOS mRNA levels were analyzed by RT-qPCR and normalized against 18S rRNA and NO levels were quantified by the Griess reaction in culture supernatants **(C)**. Results are expressed as the mean of three independent experiments (*n* = 3, 5 replicates/treatment) ± SEM. White bar: Unstimulated control cells. Black bar: LPS stimulated cells. Gray bar: LPS-stimulated and Bzl-treated cells. Hatched gray bar: SOCS3 silenced, LPS-stimulated and Bzl-treated cells. **P < 0.001, vs. LPS-stimulated cells; ^##^*P* < 0.001 vs. control cells.^ø^*P* < 0.05 vs. Bzl-treated and LPS-stimulated cells.

In order to determine whether SOCS3 participates in the inhibition of inflammatory mediators, we accomplished specific knockdown of SOCS3 with siRNA in cultured cardiomyocytes. Then, cells were pre-treated with benznidazole and stimulated with LPS. IL-6 and TNF-α expression and their concentrations in culture supernatants were evaluated. Figure 3A shows that benznidazole inhibited IL-6 and TNF-α mRNA expression. Conversely, when SOCS3 was silenced, benznidazole was unable to modulate the expression of these pro-inflammatory cytokines. In agreement with this benznidazole could not inhibit the release of IL-6 and TNF-α to culture supernatants upon SOCS3 silencing (Figure 3B). Also, under silencing conditions of SOCS3, benznidazole neither inhibited iNOS mRNA expression nor NO production in LPS-stimulated cells (Figure 3C).

### Benznidazole Inhibits IKK/NF-κB Activation Through SOCS3

**Figure 4:**
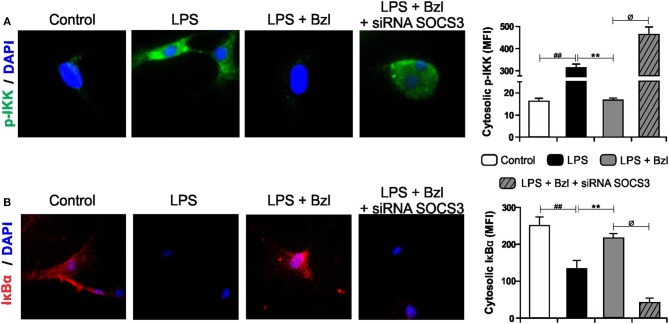
Cardiomyocytes were transfected with SOCS3 siRNA. After 72 h, cells were treated with 15 μM of benznidazole (Bzl) for 30 min and then with 10 mg/L LPS. p-IKK (15 min) **(A)**, and IκBα (30 min) **(B)** expression was detected by immunofluorescence with a rabbit polyclonal IgG anti- p-IKK or anti- IκBα (primary antibodies), and Goat anti-rabbit IgG Alexa Fluor 488 or Alexa Fluor 594 (secondary antibodies), respectively. Cell nuclei were stained with 300 nM DAPI. Representative microphotographs (400x) are shown. Mean fluorescence intensity (MFI) was quantified using Fiji Software, and the results are expressed as the mean of three independent experiments (*n* = 3, 5 replicates/treatment) ± SEM. White bar: Unstimulated control cells. Black bar: LPS-stimulated cells. Gray bar: LPS-stimulated and Bzl-treated cells. Hatched gray bar: SOCS3- silenced, LPS-stimulated and Bzl-treated cells. **P < 0.001, vs. LPS-stimulated cells; ^##^*P* < 0.001 vs. control cells.^ø^*P* < 0.05 vs. Bzl-treated and LPS-stimulated cells.

The transcription factor NF-κB is known to participate in the induction of pro-inflammatory genes. We previously showed that a low dose of benznidazole reduces the expression of inflammatory mediators by inhibiting NF-κB pathway, although the mechanism involved is poorly understood (6). Therefore, in this work we designed a set of experiments to evaluate whether SOCS3 participates in the effects of benznidazole on the NF-κB pathway. Figure 4A shows IKK phosphorylation visualized using a fluorescence microscopy upon challenge for 15 min with LPS. When benznidazole was added, IKK phosphorylation was inhibited. However, when SOCS3 was silenced, benznidazole could not inhibit IKK phosphorylation. Similar results were obtained when we evaluated cytosolic IκBα degradation. The NF-κB inhibitor, IκBα, underwent degradation upon stimulation with LPS and this effect was prevented in the presence of benznidazole. The participation of SOCS3 was confirmed, since benznidazole did not inhibit the degradation of IκBα promoted by LPS stimulation in the cells in which SOCS3 was silenced (Figure 4B).

### IL-10 Participates in Benznidazole Anti-inflammatory Effects

IL-10 is a key inhibitor of many aspects of the inflammatory response. In previous reports, we highlighted the relevance of IL-10 in the modulation of the pro-inflammatory response of cardiomyocytes in Chagas disease. We showed that recombinant mouse IL-10 acts as a negative regulator of pro-inflammatory signaling by up-regulation of SOCS3 in cardiomyocytes infected with *T. cruzi* (31). Moreover, it has been reported that peripheral blood mononuclear cells isolated from patients in the indeterminate phase of Chagas disease produce higher levels of IL-10 than those isolated from patients with chronic cardiomyopathy (49, 50).

**Figure 5:**
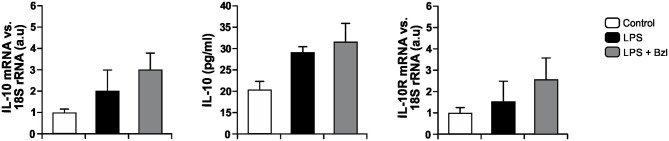
Cardiomyocytes were treated with 15 μM of benznidazole (Bzl) for 30 min and then with 10 mg/L LPS. After 24 h, IL-10 and IL-10 receptor (IL-10R) mRNA levels were analyzed by RT-qPCR and normalized against 18S rRNA. Concentration of IL-10 in culture supernatants was measured by ELISA. Results are expressed as the mean of three independent experiments (*n* = 3, 5 replicates/treatment) ± SEM. White bar: Unstimulated control cells. Black bar: LPS-stimulated cells. Gray bar: LPS-stimulated and Bzl-treated cells.

In order to establish whether IL-10 could be involved in the effects of benznidazole, we evaluated the expression of IL-10 and its receptor in LPS-stimulated and benznidazole pre-treated cardiomyocytes as well as the release of IL-10 to culture supernatants. Figure 5 shows a trend to increase the expression of IL-10 and its release to culture medium, as well as the expression of IL-10R in cardiomyocytes stimulated with LPS, and that pre-treatment with benznidazole further increases this trend.

**Figure 6:**
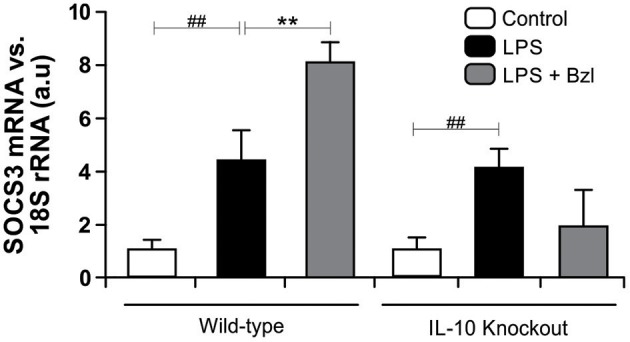
Cardiomyocytes from BALB/c and IL-10-knockout (IL-10-KO) mice of BALB/c background were treated with 15 μM of benznidazole (Bzl) for 30 min and then with 10 mg/L LPS for 24 h. SOCS3 mRNA levels were analyzed by RT-qPCR and normalized against 18S rRNA. Results are expressed as the mean of three independent experiments (*n* = 3, 5 replicates/treatment) ± SEM. White bar: Unstimulated control cells. Black bar: LPS-stimulated cells. Gray bar: LPS-stimulated and Bzl-treated cells. **P < 0.001, vs. LPS-stimulated cells; ^##^*P* < 0.001 vs. control cells.

Moreover, to determine whether benznidazole requires IL-10 for the increase of SOCS3, we evaluated the expression of SOCS3 in neonatal cardiomyocytes isolated from IL-10-knockout mice (41). Figure 6 shows that LPS increases SOCS3 mRNA expression in IL-10-knockout cardiomyocytes. However, when these cells were pre-incubated with benznidazole, its expression did not increase, strongly suggesting that benznidazole induction of SOCS3 depends on IL-10 signaling.

**Figure 7:**
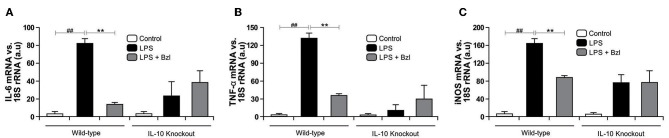
Cardiomyocytes from BALB/c and IL-10-knockout (IL-10-KO) mice of BALB/c background were treated with 15 μM of benznidazole (Bzl) for 30 min and then with 10 mg/L LPS. After 4 h, IL-6 **(A)** and TNF-α **(B)** mRNA levels were analyzed by RT-qPCR and normalized against 18S rRNA. After 48 h, iNOS mRNA levels were analyzed by RT-qPCR and normalized against 18S rRNA **(C)**. Results are expressed as the mean of three independent experiments (*n* = 3, 5 replicates/treatment) ± SEM. White bar: Unstimulated control cells. Black bar: LPS-stimulated cells. Gray bar: LPS-stimulated and Bzl-treated cells. **P < 0.001, vs. LPS-stimulated cells; ^*##*^*P* < 0.001 vs. control cells.

**Figure 8:**
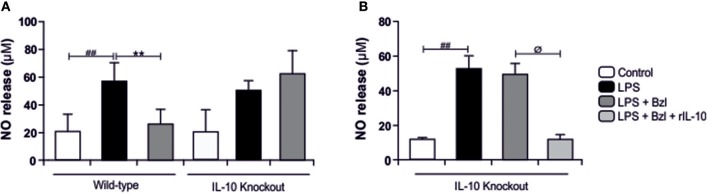
Cardiomyocytes from BALB/c and IL-10-knockout (IL-10-KO) mice of BALB/c background were treated with 15 μM of benznidazole (Bzl) for 30 min and then with 10 mg/L LPS. After 48 h NO levels were quantified by the Griess reaction in culture supernatants **(A)**. Cardiomyocytes from IL-10-KO mice were treated with 50 ng/ml of recombinant mouse IL-10 (rIL-10) for 30 min before Bzl treatment and LPS stimulation. After 48 h NO levels were quantified by the Griess reaction in culture supernatants **(B)**. Results are expressed as the mean of three independent experiments (*n* = 3, 5 replicates/treatment) ± SEM. White bar: Unstimulated control cells. Black bar: LPS-stimulated cells. Gray bar: LPS-stimulated and Bzl treated cells. Light-Gray bar: rIL-10- and Bzl-treated, LPS stimulated cells. **P < 0.001, vs. LPS-stimulated cells; ^##^*P* < 0.001 vs. control cells.^ø^*P* < 0.05 vs. Bzl treated-LPS-stimulated cells.

In the same direction we observed that benznidazole fails to modulate the expression of IL-6, TNF-α, and iNOS in cardiomyocytes lacking IL-10 (Figure 7). In addition, when we evaluated NO release to culture supernatant, the secretion induced by LPS cannot be inhibited by benznidazole in IL-10 knockout cardiomyocytes (Figure 8A), and this was restored in the presence of recombinant IL-10 (Figure 8B).

**Figure 9:**
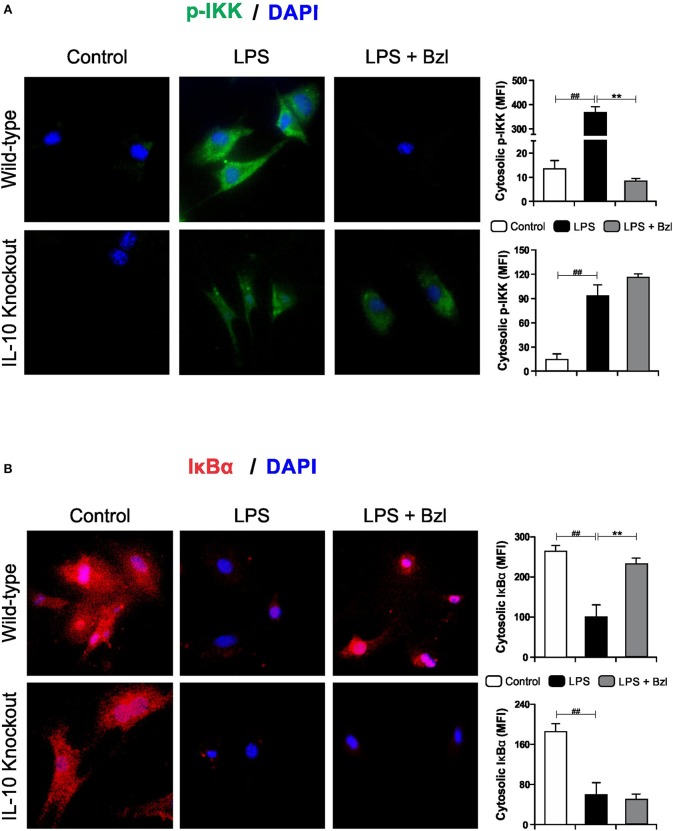
Cardiomyocytes from BALB/c and IL-10-knockout (IL-10-KO) mice of BALB/c background were treated with 15 μM of benznidazole (Bzl) for 30 min and then with 10 mg/L LPS. p-IKK (15 min) **(A)** and IκBα (30 min) **(B)** expression was detected by immunofluorescence with a rabbit polyclonal IgG anti- p-IKK or anti- IκBα (primary antibodies), and Goat anti-rabbit IgG Alexa Fluor 488 or Alexa Fluor 594 (secondary antibodies), respectively. Cell nuclei were stained with 300 nM DAPI. Representative microphotographs (400x) are shown. Mean fluorescence intensity (MFI) was quantified using Fiji Software, and the results are expressed as the mean of three independent experiments (*n* = 3, 5 replicates/treatment) ± SEM. White bar: Unstimulated control cells. Black bar: LPS-stimulated cells. Gray bar: LPS-stimulated and Bzl-treated cells. **P < 0.001, vs. LPS-stimulated cells; ^##^*P* < 0.001 vs. control cells.

Furthermore, benznidazole could not avoid the phosphorylation of IKK (Figure 9A) and degradation of IκBα (Figure 9B) in IL-10-knockout mice, as shown by immunofluorescence microscopy. This confirms that IL-10 is required for the benznidazole-mediated inhibition of NF-κB.

## Discussion

Benznidazole is one of the anti-parasitic drugs currently used in the treatment of Chagas disease. This drug has also anti-inflammatory effects, the mechanisms of which have not been fully elucidated yet. During the acute infection, the inflammatory response must be critically balanced. Although necessary for the control of parasite proliferation, inflammation results in tissue damage, leading to progressive myocardial fibrosis, and cardiac remodeling, ultimately leading to heart failure. The evolution of this disease causes major disabilities with high economic and social impact (51). For these reasons, besides controlling the parasite load, control of the inflammatory response both in the early and chronic stages of the infection is especially important.

Although benznidazole is one of the main drugs used in the treatment of acute and early chronic Chagas disease, there are serious limitations, such as the side effects exhibited by a significant proportion of patients (52). These include organic manifestations indicative of systemic toxicity. Adverse effects symptoms are evident in the first days of treatment and represent a major threat of benznidazole in clinical use with frequent therapy discontinuation (52–56). In a previous work we described, for the first time, that benznidazole has parasiticidal properties, *in vivo* as well as *in vitro*, at doses or concentrations lower than those previously reported for a highly virulent *T. cruzi* strain. In addition, treatment with a low dose of benznidazole was also able to inhibit the expression of inflammatory mediators and tissue inflammation in an experimental model of Chagas disease (6). *In vitro* studies using primary culture cardiomyocytes infected with the virulent RA strain of *T. cruzi* showed that 15 μM benznidazole was the optimal concentration to reduce the parasite load as well as the inflammatory mediators in the infected cells (6). This finding led us to search for the mechanism of action involved in the anti-inflammatory effects, knowing the optimal parasiticidal concentration and considering that this is the only antiparasitic treatment currently available in many countries.

In a previous work, we found that *T. cruzi* and IL-10 promotes STAT3 phosphorylation and up-regulates the expression of SOCS3 thereby preventing NF-κB nuclear translocation and ERK1/2 phosphorylation, two of the pathways known to promote the expression of pro-inflammatory genes (31).

In this work we focused on the pathways through which benznidazole exerts anti-inflammatory effects, demonstrating the IL-10/STAT3/SOCS3 axis is involved. To this aim we proceeded to dissociate the antiparasitic effects and anti-inflammatory properties of benznidazole using an *in vitro* model of primary culture cardiomyocytes pre-treated with benznidazole and stimulated with LPS. We report, for the first time, that benznidazole increases SOCS3 expression.

It has been described that phosphorylated STAT3 is dimerized and induces transcription of the SOCS3 gene (57). Also, it has been reported that the induction of SOCS3 expression in macrophages infected with *Mycobacterium tuberculosis* leads to the inhibition of NF-κB activation as a mechanism of immune evasion (58). Moreover, Carow et al. demonstrated that SOCS3, both in myeloid and T cells, is essential for resistance against *M. tuberculosis via* discrete mechanisms (59).

In the 1960s, it was postulated that the production of reactive oxygen species (ROS) would be one of the mechanisms capable of killing pathogens during the respiratory outbreak by phagocytes (60). In the same way, the trypanocidal activity of benznidazole, for instance, was credited to its capability to cause oxidative damage to the parasite (61). Paradoxically, it is also suggested that oxidative environments would stimulate parasite growth (62). However, it must be borne in mind that excessive ROS production and secretion to the tissue *milieu* can be detrimental to the host in the context of persistent inflammation.

Taking into consideration that *T. cruzi* can activate STAT3, and also increase SOCS3 expression (31), we wondered whether benznidazole was also able to promote STAT3 activation. In the current work we show that LPS also increases SOCS3. Moreover, the addition of benznidazole increases SOCS3 more than 50-fold with respect to LPS. Interestingly, both IL-10 and IL-6 induce the activation of STAT3, yet generate different cellular responses. While IL-6 stimulation promotes a pro-inflammatory response, IL-10 signaling induces a strong anti-inflammatory one (36). The increase in SOCS3 in LPS-stimulated cardiomyocytes might be dependent on the IL-6 pathway of STAT3 activation, since we observed a significant amount of IL-6 in the cell supernatants at 48 h. We hypothesized that up-regulation of SOCS3 in benznidazole-treated cardiomyocytes was mediated through IL-10. This was confirmed in our model using IL-10KO cardiomyocytes, in which upregulation of SOCS3 is not observed. Furthermore, the increase of SOCS3 was precluded by Stattic, suggesting that benznidazole increases SOCS3 expression in a STAT3-dependent manner. Previously, we reported that benznidazole inhibits iNOS and consequently NO release in LPS stimulated cardiomyocytes (6). In this work, we demonstrate the participation of STAT3 in the inhibitory effect of benznidazole since when STAT3 was inhibited by Stattic, benznidazole could neither inhibit the expression of iNOS nor the release of NO. Furthermore, when SOCS3 was silenced in cardiomyocytes stimulated with LPS, using SOCS3-specific siRNA, benznidazole neither inhibited the expression nor the release of inflammatory cytokines. Similar effects were observed for the expression of iNOS and release of NO to the culture medium.

It has been described that SOCS3 exerts its anti-inflammatory effects by inhibition of the activation of the NF-κB pathway (63). In this regard, it has been demonstrated that the PPE18 protein of *M. tuberculosis* up-regulates the expression as well as tyrosine phosphorylation of SOCS3. Phosphorylated SOCS3 physically interacts with the IκBα-NFκB/rel complex, inhibiting phosphorylation of IκBα and thus inactivates the NF-κB pathway (58). Moreover, we have demonstrated that knockdown of SOCS3 by specific siRNA, impedes the IL-10-mediated inhibition of NFκB and ERK1/2 activation in cardiomyocytes infected with *T. cruzi* (31). Furthermore, we previously showed that a low dose of benznidazole reduces the expression of inflammatory mediators by inhibiting the NFκB pathway (6). In this work we demonstrate the participation of SOCS3 in the effects of benznidazole, because when this cytokine suppressor was silenced in cardiomyocytes stimulated with LPS, the treatment with benznidazole could neither inhibit the IKK phosphorylation nor prevent IκBα degradation. Therefore, under conditions that preclude SOCS3 signaling, the NF-κB pathway cannot be inhibited.

Previously, we have demonstrated that the treatment with mouse recombinant IL-10, acts as a negative regulator of pro-inflammatory signaling by up-regulation of SOCS3 in cardiomyocytes infected with *T. cruzi* (31). Then, we sought to determine whether IL-10 was involved in the anti-inflammatory effects mediated by benznidazole. Our results showed, first of all, that LPS and LPS plus benznidazole increase the release of IL-10 and the expression of its receptor. Interestingly, the assays show that the increase in the expression of SOCS3 depends on the presence of IL0, since benznidazole was unable to increase the expression of SOCS3 in cardiomyocytes from IL-10-knockout mice. Secondly, benznidazole failed to inhibit the expression of inflammatory mediators in IL-10-knockout cells. There are evidences about the participation of SOCS3 in the mechanism by which IL-10 modulates the inflammatory response. In this regard, it has been reported that SOCS3 is the key mediator of the inhibitory effects of IL-10 in macrophages stimulated with LPS (64–66), and in an *in vivo* model of collagen induced arthritis (67). Moreover, our previous results highlight the relevance of IL-10 in the modulation of pro-inflammatory response in cardiomyocytes infected with *T. cruzi*, as we demonstrated that the IL-10/STAT3/SOCS3 axis negatively regulates NF-κB and ERK/MAPK–dependent gene expression of pro-inflammatory mediators (31). Furthermore, the relevance of IL-10 was evident as we demonstrated that benznidazole can neither inhibit IKK phosphorylation nor prevent the degradation of IκBα in cardiac cells from IL-10-knockout mice. Thus, IL-10 is required for the inhibitory effects of benznidazole on the NF-κB pathway to take place. Lastly, it has been widely described that the modulatory effects of IL-10 on the inflammatory response are associated with the inhibition of the NF-κB pathway (68–70), making IL-10 a reasonable mediator of benznidazole anti-inflammatory mechanisms.

**Figure 10:**
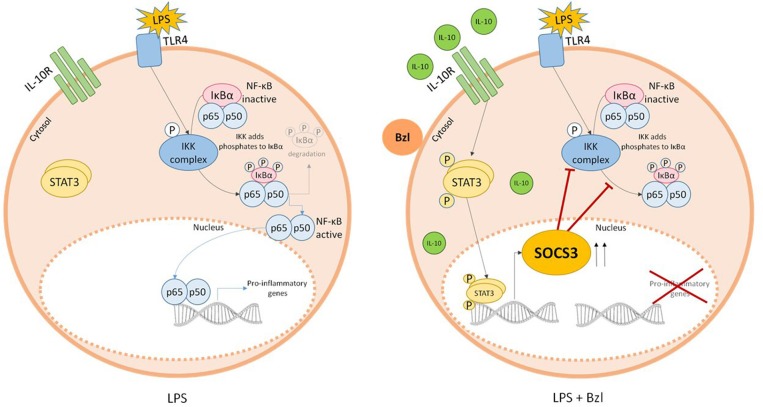
Parasiticidal concentrations of benznidazole (Bzl) exert anti-inflammatory effects through IL-10/STAT3/SOCS3 axis. When cardiomyocytes are stimulated with LPS and pre-treated with 15 μM of Bzl, IL-10 expression tends to increase. This induces the phosphorylation of STAT3 resulting in an increase in SOCS3 expression, thereby preventing NF-κB activation.

In summary, we show for the first time, that the IL-10/STAT3/SOCS3 axis is involved in the anti-inflammatory effects of benznidazole (Figure 10). These findings may add up to new therapeutic strategies for the treatment of chronic Chagas disease given both its anti-parasitic and inflammatory properties.

## Ethics Statement

To carry out this work, CF1, BALB/c, and BALB/c-background IL-10 knockout mice, homozygous for the targeted mutation Il10tm1Cgn (Stock Number 004333; The Jackson Laboratory, USA) (41) were used. All the animals were bred and maintained in the animal facility at the Instituto de Investigaciones en Microbiología y Parasitología Médica, Universidad de Buenos Aires—CONICET. All the procedures were approved by the Institutional Committee for the Care and Use of Laboratory Animals (CICUAL, Facultad de Medicina de la Universidad de Buenos Aires, CD N° 2271/2014), in line with guidelines of the Argentinean National Administration of Medicines, Food, and Medical Devices (ANMAT), Argentinean National Service of Agri-Food Health and Quality (SENASA) and with the Guide for the Care and Use of Laboratory Animals (NIH, USA).

## Author Contributions

NG and ÁC designed experiments and analyzed data. ÁC, NG, and GM contributed to the writing of the manuscript. ÁC and FP did experiments. CA provided the IL-10 knockout mice. NG, GM, FP, CA, and ÁC contributed to final approval of the version to be published.

### Conflict of Interest Statement

The authors declare that the research was conducted in the absence of any commercial or financial relationships that could be construed as a potential conflict of interest.
